# Editorial: Advances in the surgical management of gastric and colorectal cancers

**DOI:** 10.3389/fonc.2023.1292465

**Published:** 2023-10-30

**Authors:** Paola Parente

**Affiliations:** Pathology Unit, Fondazione IRCCS Casa Sollievo della Sofferenza, San Giovanni Rotondo, Italy

**Keywords:** colorectal cancer, gastric cancer, surgical procedures, endoscopy - methods, nomogram

The Research Topic ‘*Advances in the Surgical management of Gastric and Colorectal cancer*’ started in October 2022 and ended in May 2023. The aim of this project has been to describe the most recent innovations in surgical treatment and/or endoscopic techniques in localized gastric cancer (GC) and colorectal cancers (CRC) and to investigate the advances in adjuvant or neoadjuvant therapies in metastatic setting. Issues in novel biomarkers with prognostic impact were also solicited. Surprisingly (and for this we are very grateful to the Authors), in less than a year we received more than 100 submissions and 34 papers were accepted for publication, after a careful and detailed peer-review process, with a total of 230 Authors involved. In particular, 23 Original Research Articles, 7 Systematic Reviews and 4 Case Reports have been published (**Figure 1A**).

**Figure 1:**
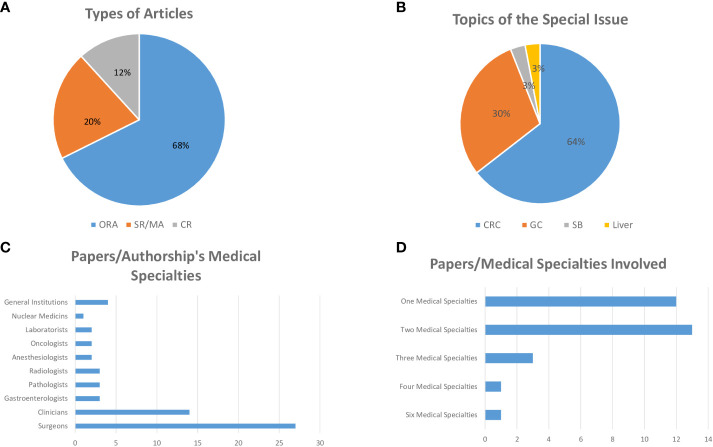
**(A)** ORA, Original Research Article; SR/MA, Systematic Review/Meta-Analysis; CR, Case Report. **(B)** CRC, Colorectal Cancer; GC, Gastric Cancer; SB, Small Bowel.

In this Editorial we aim to resume some characteristics of these papers and raise some reflections.

Among Original Research Articles concerning CRC issues, Yang et al. investigated the impact of the total number of lymph nodes retrieved on the prognosis after neoadjuvant chemo and radio therapy in 257 consecutive patients with locally advanced CRC who underwent laparoscopic surgery. Multivariate analysis showed that only patient age, Tumor Regression Grade (TRG) score and ypN stage were independent factors affecting the number of lymph nodes (P<0.05). Moreover, less than 12 lymph nodes dissected has not been proved to be an adverse predictor for long-term survival (*Lymph node yield less than 12 is not a poor predictor of survival in locally advanced rectal cancer after laparoscopic TME following neoadjuvant chemoradiotherapy*).

Wang et al. explored the efficacy of transatmospheric ileal stoma manometry (TISM) in early detection of stoma outlet obstruction (SOO) before the manifestation of intestinal obstruction in a total of 38 patients who were subjected to laparoscopic anterior rectal resection and diverting ileostomy for CRC. Group with SOO had significantly higher stoma pressure on the second day after return of gut function compared to those without SOO (p<0.001), suggesting TISM can be used as a supplementary method for the early detection of SOO and allow timely treatment of the patients before they develop symptoms of obstruction (*Transatmospheric ileal stoma manometry can be applied for the early detection of stoma outlet obstruction).*

Some Original Research Articles focused on new surgical approaches in CRC. In particular, Ren et al. explored the short-term and long-term efficacy of superior rectal artery (SRA) preservation in laparoscopic radical resection for sigmoid cancer. A total of 84 patients received lymph node clearance around the inferior mesenteric artery (IMA) root (D3 lymph node dissection) with preservation of SRA (SRA preservation group), and 123 patients received high ligation of the IMA (control group). The results indicated that SRA preservation does not increase postoperative morbidity and mortality, does not affect prognosis, and can achieve D3 lymph node dissection, which is technically completely feasible (p<0.05) a front of the operation time, significantly longer in the preservation group (p<0.001) (*Feasibility of preservation of superior rectal artery plus dissection of lymph nodes around inferior mesenteric artery in laparoscopic resection for sigmoid colon cancer*).

Lu et al. described suitability of laparoscopic modified Bacon in 60 patients in stage I-III low rectal cancer. In particular, this new approach is safe for older patients or those with high risk factors for anastomotic leakage such as diabetes, chronic obstructive pulmonary disease, and hypoproteinemia (P <0.05) respect to the control cases. This technique can improve the rate of anal preservation, and save for rectal benign tumors or other rectal tumors that cannot be locally resected (e.g., large villous adenoma of the rectum, rectal gastrointestinal stromal tumor) (*Application of laparoscopic modified Bacon operation in patients with low rectal cancer and analysis of the changes in anal function: A retrospective single-center study*).

Liu et al. investigated the effect of carbon nanoparticles staining (CNS) in 6886 CRC resected patients, on lymph node tracing and postoperative complications using propensity score matching (PSM). PSM from age, sex, body mass index (BMI), smoking, drinking, hypertension, type 2 diabetes mellitus (T2DM), coronary heart disease (CHD), surgical history, open surgery, tumor location, tumor nodes metastasis (TNM) stage, tumor size and CNS suggested that CNS before surgery could help surgeons to retrieve more lymph nodes (P <0.001), reduce intraoperative blood loss (P =0.004) and reduce hospital stay (P <0.001) respect to non-CNS group (*The effect of carbon nanoparticles staining on lymph node tracking in colorectal cancer: A propensity score matching analysis*).

Qiu et al. compared safety and feasibility of advance incision (AI) technique for robotic-assisted laparoscopic rectal anterior resection in 102 patients. The mean time to perform auxiliary incisions and the average incision length were shorter (p<0.05) in the AI group with respect to the control group. No significant differences in incision infection, incision hematoma, incision healing time, and long-term incision complications, including incision hernia and intestinal obstruction, were observed between groups, supporting the utility of AI technique (*Application of the advance incision in robotic-assisted laparoscopic rectal anterior resection).*

Chen et al. applied indocyanine green (ICG) angiography to prevent anastomotic leakage (AL) in rectal cancer in 286 patients. Less patients in the ICG group were diagnosed with AL respect to non-ICG (p<0.001). Moreover, the ICG group had a less hospital readmission rate than the non-ICG group (p=0.003), showing ICG can reduce AL and hospital readmission rates (*Indocyanine green angiography for lower incidence of anastomotic leakage after transanal total mesorectal excision: a propensity score-matched cohort study*).

Zhou et al. investigated intracorporeal anastomosis (IA) approach for reconstruction of digestive tract after laparoscopic right hemicolectomy with respect to extracorporeal anastomosis (EA) in 78 patients with CRC. Although the postoperative complication rate of IA is similar to that of EA, the intraoperative blood loss was less (P =0.010) and the incision length was shorter (P <0.001) in the IA group. Moreover, postoperative farting time was faster (P =0.005) and postoperative pain score (VAS) was lower (P <0.001) in the IA group. These results support IA as an alternative of EA in high volume surgical centers, after adequate technical training (*Intraoperative and postoperative short-term outcomes of intracorporeal anastomosis versus extracorporeal anastomosis in laparoscopic right hemicolectomy*).

Yu et al. investigated transvaginal natural orifice specimen extraction surgery (NOSES) in the right hemicolectomy with respect to traditional transabdominal specimen extraction surgery in 138 CRC patients. Transvaginal NOSES showed better short-term outcomes than transabdominal specimen extraction surgery (p<0.001), such as less intraoperative bleeding and faster recovery of gastrointestinal function. Moreover, transvaginal NOSES provided a better postoperative quality of life and scar satisfaction suggesting this new approach as a valid alternative (*Transvaginal versus transabdominal specimen extraction surgery for right colon cancer: A propensity matching study*).

Yang et al. explored the safety of transanal total mesorectal excision (TaTME) with respect to laparoscopic transabdominal total mesorectal excision (TME) in 51 patients with low rectal cancer. The incidence of postoperative complications in the TaTME group was significantly lower than that in the TME group (*P*>0.05), without significant difference in operation time (*P*>0.05), suggesting TaTME resection as safe and effective alternative to laparoscopic transabdominal TME *(Simple transanal total mesorectal resection versus laparoscopic transabdominal total mesorectal resection for the treatment of low rectal cancer: a single-center retrospective case-control study).*

Concerning the new approaches in GC surgery, Park et al. led a prospective single-arm open-label observational study to investigate the feasibility of a patient-specific 3-D surgical navigation system for preoperative planning and intraoperative guidance during robotic surgery in 30 patients with GC. Results showed in the experimental group a shorter anesthesia time (*P*=0.299), operative time (*P* =0.137), and console time (*P* =0.101) than the control group, validating patient-specific 3-D surgical navigation system as clinically feasible and applicable with an acceptable turnaround time (*Patient-specific virtual three-dimensional surgical navigation for gastric cancer surgery: A prospective study for preoperative planning and intraoperative guidance*).

Shen et al. investigated robotic distal gastrectomy (RDG) in 110 patients with stage I-III GC. RDG showed a comparable operation time and lower volume of blood loss compared with laparoscopic distal gastrectomy (LDG) if performed by surgeons stepping into the stable stage of the robotic learning curve (*P*=0.434) (*Comparison of short-term outcomes between robotic and laparoscopic distal gastrectomy performed by the same surgical team during the same period*).

Jin et al. performed normothermic intraoperative intraperitoneal chemotherapy (NIIC) in a total of 1253 patients with *naive* stage I-III GC, showing no significant difference in overall survival (*P*>0.05). The multivariate Cox analysis confirmed that only age, BMI, pathological type, TNM stage, and differentiation grade were independent risk factors of survival in GC (*A real-word study: is normothermic intraoperative intraperitoneal chemotherapy impactful as we expect*)*?*.

Ye et al. explored laparoscopic digestive tract nutrition reconstruction (LDTNR) combined with conversion therapy (epirubicin + oxaliplatin + capecitabine regimen) in improving the inflammatory nutritional immune status and survival time in 37 patients with unresectable advanced GC with obstructive symptoms. The median chemotherapy cycle of patients in LDTNR group was higher than that in non-LDTNR group (P <0.001), with a significantly better response rate and 3-year cumulative survival rate respect to non-LDTNR group (P <0.001) (*Safety and efficacy of laparoscopic digestive tract nutrition reconstruction combined with conversion therapy for patients with unresectable and obstructive gastric cancer*).

Other Original Research Articles explored predictive usefulness of nomograms application. In detail, Huang et al. proposed a nomogram including preoperative triglyceride/high-density lipoprotein cholesterol (TG/HDL-C) ratio (THR) ≥ 1.93 and prognostic nutritional index (PNI) ≥ 42.55 as independently associated with overall survival (OS) and cancer-specific survival (CCS) in 523 stage I-III CRC patients. In particular, six (pT stage, pN stage, histological subtype, perineural invasion, THR and PNI) and seven (pT stage, pN stage, histological subtype, perineural invasion, gross appearance, THR and PNI) variables were chosen to develop nomograms for the prediction of OS and CCS. The nomograms incorporating the two indexes provide a reliable approach for predicting the prognosis and optimizing individualized therapy of non-metastatic CRC patients (*Prognostic nomograms integrating preoperative serum lipid derivative and systemic inflammatory marker of patients with non-metastatic colorectal cancer undergoing curative resection*).

Su et al. investigated a nomogram to predict cancer-specific survival (CSS) of early-onset locally advanced rectal cancer (EO-LARC) in 2440 patients from the Surveillance, Epidemiology, and End Results (SEER) database. Seven variants (sex, pathology, radiotherapy, grade, T-stage, CEA, and LNR) have been shown to be independent risk factors affecting the postoperative outcome of EO-LARC patients and taken in consideration. This nomogram is able to predict CSS at 3, 6, and 9 years in patients with EO-LARC. The coherence index (C-index), net reclassification index (NRI), ROC, and integrated discrimination improvement (IDI) demonstrated that the nomogram had better clinical value than AJCC staging (*Early-onset locally advanced rectal cancer characteristics, a practical nomogram and risk stratification system: a population-based study*).

Li et al. described a nomogram predicting the risk of lymph nodes metastasis (LNM) in 626 early GC (EGC) patients according to proportions of undifferentiated components (PUC), histologically detected. Compared with the pure differentiate group (PD), LNM rate was higher in group M4 (60%<PUC ≤ 80%) and group M5 (80%<PUC < 100%) (P < 0.05). Differences of tumor size, presence of lymphovascular invasion (LVI), perineural invasion and invasion depth also exist between groups. Multivariate analysis revealed that tumor size over 2 cm, submucosal invasion, presence of LVI and PUC level M4 significantly predicted LNM in EGC (*Clinicopathological characteristics of early gastric cancer with different level of undifferentiated component and nomogram to predict lymph node metastasis*).

One Original Research Article Original faced radiological issues in CRC. Wang et al. demonstrated delta-Radiomics model was superior to the pre-Radiomics model in predicting the treatment response to neoadjuvant chemoradiotherapy (NCRT) in 84 patients with locally advanced CRC *(MRI-based pre-Radiomics and delta-Radiomics models accurately predict the post-treatment response of rectal adenocarcinoma to neoadjuvant chemoradiotherapy)*.

Finally, Original Research Articles describing serological markers/pathological features utility in predicting outcome have been included. Costantini et al. evaluated a large panel of cytokines, chemokines, and growth factors in the plasma after bevacizumab plus oxaliplatin-based regimens within the Obelics trial (NCT01718873) in a cohort of 30 metastatic CRC (mCRC) patients undergoing curative resection of liver metastases. Notably, metabolite-set enrichment analysis, evaluated in plasma at the time of response evaluation before surgery, highlighted a complex interplay between different metabolic pathways that clearly distinguished poor *vs* good outcome patients suggesting a potential role of these combined biomarker in defining personalized management and treatment strategies (*Plasma metabolomics, lipidomics and cytokinomics profiling predict disease recurrence in metastatic colorectal cancer patients undergoing liver resection*).

Concerning GC, Du et al. investigated the predictive ability of the PNI-IgM score (a combined prognostic nutritional index (PNI), and immunoglobulin M (IgM)) on the prognosis in 340 patients undergoing surgery for GC. The score 1 (the lower the PNI-IgM score; PNI< 48.45 and IgM < 0.87) has been associated with the worse prognosis, suggesting this novel combination of nutritional and immunological markers can be used as a sensitive biological marker for patients with GC who undergo surgery (*Combined with prognostic nutritional index and IgM for predicting the clinical outcomes of gastric cancer patients who received surgery).*

Liu et al. investigated the impact of type 2 diabetes mellitus (T2DM) on the short-term outcomes and long-term survival in 272 CRC patients (136 patients for T2DM group and 136 for non-T2DM group). Higher body mass index (BMI), higher proportion of hypertension and cerebrovascular diseases (*P*<0.05), more overall complications (*P* =0.001), and higher risk of re-operation (*P* =0.007) were found in T2DM group when compared to the control group. Moreover, T2DM patients had longer hospitalization time and worse 5-year overall survival (*P* =0.024) and 5-year disease-free survival (DFS) (*P* =0.019) in all stage (*Marked paper: Type 2 diabetes mellitus indicates increased postoperative complications and poor prognosis in colorectal cancer patients receiving curative surgery*).

Omeroglu et al. have explored the predictive value of metastatic lymph node (MLN) size on prognosis and survival in 101 stage III CRC patients who underwent curative resection. Among the patients with MLN diameter ≥ 1.05 cm, the maximum size of tumor was larger and number of MLNs were higher than those of patients with MLN diameter < 1.05 cm (p=0.049 and p<0.001, respectively), suggesting that histopathological measurement of MLN size may contribute to predicting the prognosis with a MLN larger than 1.05 cm being predictive for a poor prognosis and lower survival (*Clinical significance of the histopathological metastatic largest lymph node size in colorectal cancer patients*).

Yao et al. investigated the clinical impact of small para-aortic lymph node (smaller than 10 mm in diameter, sPAN) in a total of 667 consecutive patients with GC resectable (98 patients in the sPAN group, and 569 patients without enlarged para-aortic lymph node in the nPAN group) in which standard D2 lymphadenectomy was performed. As cN stage has been proven to be significantly related to sPAN (p=0.001), standard D2 lymphadenectomy should be considered a valid approach in these patients. Moreover, sPAN associated to elevated CEA or CA19-9 levels has been suggested to benefit from a multimodal approach (neoadjuvant chemotherapy; radical surgery with D2 plus lymph nodal dissection extended to n°16 station) (*Clinicopathological characteristics and treatment outcome of resectable gastric cancer patients with small para-aortic lymph node).*

Concerning meta-analyses and Systematic Review, Yang et al. started a protocol for a systematic review and meta-analysis concerning effectiveness and safety of laparoscopic Transversus abdominis plane block (TAPB) compared to ultrasound-guided TAPB for postoperative pain control and opioid consumption in patients undergoing major colorectal surgeries (*Laparoscopic versus ultrasound-guided transversus abdominis plane block for postoperative pain management in minimally invasive colorectal surgery: a meta-analysis protocol*), wherease Zhang et al. launched a protocol comparing the surgical and oncological outcomes of local excision (LE) and radical excision (RE) for rectal gastrointestinal stromal tumors (GISTs) *(Local Excision and Radical Excision for Rectal Gastrointestinal Stromal Tumors: A meta-analysis protocol).*

Yan et al. compared a short- and long-term outcomes of laparoscopic distal gastrectomy (LDG) respect to open distal gastrectomy (ODG) in patients with advanced gastric cancer (AGC) in randomized controlled trials (RCTs) when performed by experienced surgeons in hospitals contending with high patient volumes. The Authors underline a certainty of evidence racing from moderate to very low; however, LDG has been showed a similar short-term surgical outcome and a long-term survival to ODG in surgical high volume Centers (*Laparoscopic vs. open distal gastrectomy for locally advanced gastric cancer: A systematic review and meta-analysis of randomized controlled trials*).

Guo et al. focused on the potentially prognostic value of the platelet-to-lymphocyte ratio (PLR) in CRC patients, showing that higher PLR levels had worse Overall Survival (*P* < 0.00001), Disease Free Survival (*P* = 0.01) and Relapse Free Survival (*P* = 0.005) than lower PLR levels, respectively. An elevated PLR seems to be an adverse prognostic factor affecting survival outcomes in patients with CRC *(Potential impact of platelet-to-lymphocyte ratio on prognosis in patients with colorectal cancer: A systematic review and meta-analysis).*

Bai et al. investigated the effectiveness of primary tumor resection (PTR) in improving the median overall survival (OS) and quality of life in patients with metastatic CRC. This review underlines all misunderstanding and bias in the studies concerning PTR, wishing randomized clinical trial to compare its effectiveness with alternative treatment options (*Primary tumor resection in colorectal cancer patients with unresectable distant metastases: a minireview).*

Wang et al. described safety of CRC surgery in the pre-pandemic and during the COVID-19 pandemic periods. Although the preoperative waiting time related to CRC surgery has been higher during the COVID-19 pandemic (p<0.00001), there was no difference in the postoperative complications, postoperative mortality, anastomotic leakage, and 30-day readmission times between pre-COVID-19 pandemic and during the COVID-19 pandemic periods. The Authors concluded that the COVID-19 pandemic did not affect the safety of CRC surgery *(The safety of colorectal cancer surgery during the COVID-19: a systematic review and meta-analysis).*

Yu et al. compared perioperative parameters, oncologic findings, and short-term postoperative outcomes between robotic gastrectomy (RG) and laparoscopic gastrectomy (LG) performed in obese patients with GC. Whereas no significant differences were observed between RG and LG groups in terms of complications, bleeding, or lymph node dissection, RG had a longer procedure time (*P*=0.03), earlier oral intake (*P*=0.0010), shorter hospital stay (*P*=0.0002), and shorter time to defecation (*P*<0.00001) (*Robotic versus laparoscopic gastrectomy for gastric cancer in patients with obesity: systematic review and meta-analysis*).

Finally, four case reports have been published, describing interesting surgical experience. Zhu et al. described laparoscopic-assisted distal gastrectomy for GC in a patient with situs inversus totalis with a detailed surgical technique description (*Case report: Laparoscopic-assisted distal gastrectomy for gastric cancer in a patient with situs inversus totalis*).

Jiang et al. run into a giant abdominal hemangioma originating from the liver with a detailed review of the Literature, suggesting a combination of imaging methods such as ultrasound, Computed Tomography, and/or Magnetic Resonance Imaging is essential for accurate diagnosis and to avoid complications in surgery *(Case Report: Giant abdominal hemangioma originating from the liver).*

Ouyang et al. reported a case of advanced rectal cancer (pT4bN2aM1b, stage IV) with the *KRAS G12D* mutation and a total of seven surgeries for relapses and long-term FOLFIRI- or XELOX-based chemotherapy regimens, with the targeted agents bevacizumab and regorafenib. The patient has been alive for 86 months since her diagnosis (*Case Report: Long-term survival of a patient with advanced rectal cancer and multiple pelvic recurrences after seven surgeries*).

Finally, Han et al. described a case of breast and vulvar metastases from rectal signet ring cell carcinoma (SRCC) with clinical and therapeutic implications (*Case Report: Systemic treatment for breast and vulvar metastases from resected rectal signet ring cell carcinoma*).

As evidence of interest about these issues, at now (end of August), in total more than 6.500 downloads have been registered.

Behind the scientific soundness of this Research Topic, it would be interesting to focus on some aspects. First: 22/34 papers (64.5%) describe issues in CRCs; 10/34 (29.5%) papers describe issues in GCs; 1/34 paper (3%) describes issue in small bowel and 1/34 (3%) in liver cancer (**Figure 1B**). Second: 33/34 papers were developed by Asian Authors and only 1 paper from Western Authors. Third: in 27/34 papers, Surgeons contributed substantially with names in the Authorship; others medical expertise were involved in the Authorship respectively as: Clinicians in 14 papers; Gastroenterologists, Pathologists, Radiologists in 3 papers; Anesthesiologists, Laboratorists, Oncologist in 2 papers; Nuclear Medicine Specialist in 1 paper. In 4 papers, the Authorship referred to general Institutions/Research Centers, without specific affiliations (**Figure 1C**). Investigating Materials and Method of Original Researches, however, in some papers are described in detail procedures and/or assays, included in the study protocols and finalized to the Results, performed by other Specialists resulting not included in the Authorship. Fourth: out of 30 papers with tracked affiliations, 1 paper (from Italian group) resulted from collaboration of six medical specialties; different contributions resulted as: four medical specialties in 1 paper; three medical specialties in 3 papers; two medical specialties in 13 papers; only one medical specialties in 12 papers (**Figure 1D**).

From the description of these data, we can draw some observations.

First of all, these papers, supported by an adequate statistical analysis, confirm and highlight a solid and continuous growth of skills and innovations in surgical and endoscopic techniques in Asian countries. Second: the higher prevalence of papers concerning CRC surgical issues, a front of a more complexity in GC surgical technique, has a socio-demographic significance. In fact, the higher incidence of gastric, esophagogastric region and esophageal cancers in Asian regions in the past decades is well known. The screening and early diagnosis with adequate endoscopic treatment developed and applied have documented a significant decrease in cases requiring surgical therapy and/or adjuvant treatment. We hope that the attention now paid to CRC can lead to increasing and improving its approaches, still today burdened by high morbidity and mortality. Finally, these studies focused on surgical techniques mentioning the clinical-pathological counterpart. Deal the GC and CRC need a multi-disciplinary approach involving many clinical figures to protect every aspect of the treatment of the ‘single’ patient at every step. Having achieved high scientific knowledge is an asset for all Researchers; expanding and involving all medical specialties lead to applying these advances in clinical practice, avoiding these studies remain an isolated experience.

## Author contributions

PP: Writing – original draft, Writing – review & editing.

